# Cartilage oligomeric matrix protein is involved in human limb development and in the pathogenesis of osteoarthritis

**DOI:** 10.1186/ar1922

**Published:** 2006-03-15

**Authors:** Sebastian Koelling, Till Sebastian Clauditz, Matthias Kaste, Nicolai Miosge

**Affiliations:** 1 Zentrum Anatomie, Abt. Histologie, Georg-August-Universitaet, Kreuzbergring 36, 37075 Göttingen, Germany

## Abstract

As a member of the thrombospondin gene family, cartilage oligomeric protein (COMP) is found mainly in the extracellular matrix often associated with cartilage tissue. COMP exhibits a wide binding repertoire and has been shown to be involved in the regulation of chondrogenesis *in vitro*. Not much is known about the role of COMP in human cartilage tissue *in vivo*. With the help of immunohistochemistry, Western blot, *in situ *hybridization, and real-time reverse transcription-polymerase chain reaction, we aimed to elucidate the role of COMP in human embryonic, adult healthy, and osteoarthritis (OA) cartilage tissue. COMP is present during the earliest stages of human limb maturation and is later found in regions where the joints develop. In healthy and diseased cartilage tissue, COMP is secreted by the chondrocytes and is often associated with the collagen fibers. In late stages of OA, five times the COMP mRNA is produced by chondrocytes found in an area adjacent to the main defect than in an area with macroscopically normal appearance. The results indicate that COMP might be involved in human limb development, is upregulated in OA, and due to its wide binding repertoire, could play a role in the pathogenesis of OA as a factor secreted by chondrocytes to ameliorate the matrix breakdown.

## Introduction

Cartilage oligomeric protein (COMP) is a protein of the extracellular matrix and can be found in human articular cartilage [1], meniscus [2], and cruciate ligament and tendon [3]. Lower concentrations of COMP can also be detected in hyaline cartilage of the human rib and trachea [4]. It has also been extracted from animal skeletal tissues, such as bovine tendon and mouse, rat, and porcine cartilage [5]. COMP is an anionic, approximately 550-kDa disulfide-linked pentameric glycoprotein and, as a member of the thrombospondin gene family, is also called thrombospondin 5 [6]. Epidermal growth factor-like and calcium-binding repeats are located in the central region of the protein [7]. The function of COMP is still not completely understood, but it binds to chondrocytes *in vitro *[8]. COMP has been shown to bind to matrilins [9] and collagen types I, II, and IX [10,11]. In contrast, COMP has no affinity to the other members of the thrombospondin family [12]. The DNA-binding protein SP1 regulates COMP expression [13] and also mechanical compression of chondrocytes [14]. COMP expression has been shown to be inhibited by leukemia/lymphoma-related factor (LRF) [15]. The human *COMP *gene is located on chromosome 19 [7]. Mutations of this gene can cause pseudoachondroplasia and multiple epiphysial dysplasia [16-18]. Furthermore, COMP has been shown to be upregulated after traumatic knee injury [19] and has been implicated in the pathogenesis of rheumatoid arthritis [20] and osteoarthritis (OA) [12,21]. During mouse development, COMP staining has been described around maturing articular chondrocytes [22], and during rat development it has been associated mainly with the growth plate [23]. Fang and colleagues [24] detected COMP as early as day 10 in murine development in the condensing mesenchyme, and later it was found in the growth plate and superficially in the developing joint cartilage. At the time of birth, COMP has been detected in the perichondrium, the periosteum, and the hypertrophic zone of mouse cartilage. This, as well as *in vitro *experimental evidence [25], has suggested that COMP is indispensable for cartilage development, but in contrast COMP knockout mice do not show an obvious skeletal phenotype [26]. There are no published results on the role of COMP during human embryonic development. A single 21-week-old human foetus has been investigated for COMP [27]. We therefore aimed to localize COMP during embryonic human limb development, describe it in adult healthy articular cartilage, and then compare its occurrence in healthy cartilage with that of diseased cartilage from late stages of OA.

## Materials and methods

### Sources of tissues

Aborted human embryos were obtained according to the regulations of the Ethics Committee of the Medical Faculty of the University of Göttingen, Germany. The embryos were classified as follows: three embryos of gestational week (gw) 8, three embryos of gw 10, and three embryos of gw 12. The ages were determined from histological data according to Carnegie stages [28]. No malformations or anomalies were observed in these specimens.

Adult human articular cartilage from the knee joint was obtained from 12 patients (ages 55–75) with OA who were undergoing total knee replacement operations. The patients met the American College of Rheumatology classification criteria for OA of the knee [29]. All patients gave their written informed consent according to the Ethics Regulations of the Medical Faculty of the Georg-August-University Göttingen. Four healthy control cartilage samples from accident victims (ages 31–50) were also investigated.

### Fixation and preparation of tissues

The abortion material and the cartilage specimens were transported to the laboratory in histidine-tryptophane-ketoglutarate solution at 4°C to ensure good preservation of the tissues [30]. The samples were fixed in 4% paraformaldehyde in phosphate-buffered saline (PBS), pH 7.4, at 4°C overnight. Bone-containing samples were decalcified with buffered EDTA for 14 days. For light microscopy, specimens were dehydrated, embedded in paraffin wax, and cut with a Reichert's microtome. For the staging of the embryos, every fifth section was stained with hematoxylin and eosin. Longitudinal sections of the cartilage specimens stained with Alcian blue were classified as stage IV according to the OA grades (I – IV) proposed by Collins and McElligott [31] in the case of the 12 patients and classified as age-dependent healthy in the case of the control cartilage samples. None of the cartilage specimens showed any signs of rheumatoid involvement or exhibited any osteophytes. From the 12 patients, cartilage samples from the deep cartilage zones near the tidemark were obtained from two different regions of the OA knee joints. One sample, with a macroscopically normal appearance, was taken from the lateral aspect of a condyle. The other one was taken from the area adjacent to the main defect at a maximum of 0.5 cm away. All cartilage specimens were also processed for ultrastructural analysis. Samples (1 mm^3^) were embedded in LR-Gold^® ^(London Resin Company, Berkshire, England) according to standard procedures, and ultra-thin sections were cut with a Reichert's ultramicrotome and collected on nickel grids coated with Formvar^® ^(Serva, Heidelberg, Germany).

### Sources of antibodies

The anti-COMP antibody is a polyclonal rabbit-anti-bovine antibody that has been affinity-purified [1]. Affinity-purified sheep-anti-digoxigenin (DIG) antibodies were purchased from Quartett (Berlin, Germany), an anti-DIG peroxidase labeled antibody from Dakopats (Hamburg, Germany), and the secondary antibodies from Medac (Hamburg, Germany).

### Samples for immunoblotting and electrophoresis

Healthy cartilage and OA cartilage samples from the area adjacent to the main defect were pulverized. Proteins were extracted using 5 M guanidine hydrochloride and protease inhibitors NEM (N-ethylmaleimide), EDTA, benzamidine hydrochloride, and amino caproic acid, precipitated in ethanol, washed in PBS, precipitated again, and finally dissolved in PBS containing 0.4% SDS. All experiments were carried out under reducing and denaturing conditions. Protein separation was performed applying SDS-PAGE and using systems containing 6% acrylamide in stacking gels and 12% in the separation gel. Tris-glycine was applied as electrophoresis buffer, and separation was carried out at 100–120 V.

### Western blot

After the electrophoresis, the proteins were blotted onto nitrocellulose membranes. Transfer was controlled by Ponceau S staining. Thereafter, membranes were washed until no color was left and then blocked overnight in PBS + 10% (w/v) milk powder at room temperature. Immunoreactions were performed applying the anti-COMP antibody for 2 hours, diluted 1:100 in PBS. The secondary goat-anti-rabbit antibody coupled to alkaline phosphatase was diluted 1:500 and incubated for 1 hour at room temperature. Three 5-minute washes with PBS were carried out between all incubation steps. Visualization was achieved using NBT/BCIP (nitrobluetetrazoline chloride/5-bromo-4-chloro-3-indolyl toluidine) coloring agent (Roche, Heidelberg, Germany).

### Light microscopic immunohistochemistry

Immunoperoxidase staining was performed on paraffin-embedded tissue sections as follows: The tissues were deparaffinized, rehydrated, and rinsed for 10 minutes in PBS. Endogenous peroxidase was inhibited by a 45-minute treatment with a solution of methanol and 3% H_2_0_2 _in the dark. Each of the reactions was followed by rinsing for 10 minutes in PBS. The sections were pre-treated for 5 minutes with 10 μg/ml protease XXIV (Sigma, Deisenhofen, Germany). The anti-COMP antibody was applied at a dilution of 1:100 in PBS for 1 hour at room temperature. A standard peroxidase-anti-peroxidase procedure followed, applying a peroxidase-coupled goat-anti-rabbit antibody (Dako, Hamburg, Germany) at a dilution of 1:150 in PBS for 1 hour at room temperature. The color reaction was carried out with DAB (diaminobenzidine) substrate (Sigma).

### Controls

As negative controls, each immunoreaction was accompanied by a reaction omitting the primary antibodies and applying rabbit serum diluted 1:100 in PBS instead. All controls proved to be negative.

### Immunogold histochemistry

As secondary antibody, an anti-rabbit immunoglobulin G (IgG) (Medac) was labeled with gold particles according to standard procedures. Ultrathin tissue sections were incubated with the anti-COMP antibodies diluted 1:100 in PBS for 16 hours at room temperature. The secondary gold-coupled antibodies, diluted 1:300 in PBS, were applied for 20 minutes at room temperature. Staining with uranyl acetate followed, and reactions were examined with the help of a Zeiss EM Leo 906E electron microscope (Carl Zeiss, Jena, Germany).

### Controls

The grids were incubated with pure gold solution in order to exclude unspecific binding of free colloidal gold. Furthermore, the reactions were performed with gold-coupled goat-anti- rabbit IgG, omitting the primary antibody to exclude non-specific IgG binding.

### Probe preparation

RNA was isolated as described below and reverse-transcribed into COMP-specific cDNA. Polymerase chain reaction (PCR) was performed with primers specific for COMP (forward AGGGAGATCGTG CAGACAA and reverse AGCTGGAGCTGTCCTGGTAG) to generate a 154 bp product. They were designed with the help of the primer^3^shareware [32]. Corresponding primers with the appropriate SP6/T7 promoter sequences were applied. *In vitro *transcription of non-radioactive sense and antisense RNAs with a DIG labeling kit (Boehringer DIG-RNA labeling kit, Boehringer, Mannheim, Germany) was performed applying SP6- and T7-polymerases (Gibco/BRL, Heidelberg, Germany). After extraction of the probes with phenol-chloroform, these were precipitated with absolute ethanol and the pellet was dissolved in DEPC-H_2_O (diethyl-pyrocarbonate).

### Light and electron microscopic *in situ *hybridization

For light microscopic investigations, paraffin sections were deparaffinized, rehydrated, and pre-treated with proteinase K. The probe concentration was 100 ng of DIG-labeled antisense probes in 100 μl hybridization solution (50% formamide, 5 × SSC, 1 μg/μl yeast-RNA, 10 ng/μl probe) for each section. Hybridization was carried out for 18 hours at 45°C. Posthybridization treatment included a washing procedure with 2 × SSC (3 × 5 minutes, at 50°C), 1 × SSC (1 × 5 minutes, at 60°C), 0.1 × SSC (1 × 15 minutes, at 60°C) and 0.05 × SSC (1 × 15 minutes, at 60°C). Afterward, the incubation with the anti-DIG peroxidase-labeled antibody diluted 1:300 in PBS was started. Finally, color reactions were started with AEC (3-amino-9-ethylcarbazol) substrate. For electron microscopy, nickel grids were incubated for 19 hours at 50°C with the same hybridization solution as described above. The probe concentration was 100 ng of DIG-labeled antisense probes in 20 μl hybridization solution per grid. Rinsing steps were the same as described above. Afterward, sections were incubated with a gold-coupled anti-DIG antibody in PBS (diluted 1:60) for 1 hour at room temperature. The specimens were rinsed with PBS, contrasted, and analyzed with the Zeiss EM Leo 906E.

### Controls

Each of the hybridizations was accompanied by one with an equivalently labeled amount of sense probe. Furthermore, hybridizations were performed without any RNA probes. Additionally, for the ultrastructural controls, tissue sections were treated with pure gold solution or the coupled anti-DIG antibody alone.

### Statistical analysis

For *in situ *hybridization at the ultrastructural level, randomly chosen micrographs of cartilage tissue with a normal appearance which were taken from the lateral aspects of a condyle and tissue samples taken from the area adjacent to the main defect from OA cartilage (*n *= 10) were pooled and counted for gold particle contents. Mean values of the numbers of gold particles per cell were analyzed in the area of 5,000 nm^2 ^in 10 cells from each patient. Significant differences in the number of gold particles were noted for *p *values (*p *≤ 0.01), using the Wilcoxon-Mann-Whitney test for unpaired samples.

### RNA extraction and real-time RT-PCR

Pieces (2 mm thick) of OA cartilage tissue taken from the area adjacent to the main defect and pieces of tissue with a macroscopically normal appearance of the lateral aspect of a condyle from each of the 12 patients were minced, and RNA was isolated according to a protocol combining Trizol^® ^and RNeasy kit (RNeasy^® ^Mini Kit, Qiagen, Hilden, Germany), following the manufacturer's instructions, and then treated with DNAfree^® ^(Ambion, Austin, TX, USA). The quality of the RNA was tested with an Agilent 2100 Bioanalyser RNA chip (Agilent, Böblingen, Germany). The RNA was reverse-transcribed with the help of the Advantage^® ^RT-for-PCR kit (BD Biosciences, San Diego, CA, USA) by applying Moloney Murine Leukemia Virus reverse transcriptase and oligo-(dT)_18_-primer.

PCR conditions were optimized by applying the gradient function of the DNA engine Opticon™ 2 (Bio-rad, München, Germany) for *HPRT-1 *(NM_000194) as housekeeping gene and for COMP. The PCR was performed in a total volume of 50 μl with 150 ng cDNA, 5 μl 10× reaction buffer, dNTP 10 μmol each, 20 pmol of each primer, and 2.5 U HotStarTaq^® ^DNA polymerase (Qiagen) with the DNA engine Opticon™ 2. After an initial activation step of 15 minutes at 95°C, further steps were as follows: 35 cycles of denaturing 30 seconds at 94°C, annealing 30 seconds at 61°C, elongation for 30 seconds at 72°C, and (lastly) extension of 10 minutes at 72°C. Ten microlitres of each sample were loaded onto a 1.5% agarose gel and were visualized by ethidium bromide after electrophoresis.

To optimize the real-time reverse transcription (RT)-PCR conditions for quantification, the optimal MgCl_2 _concentration was determined. Twelve point five microlitres of 2xQuantiTect™ SYBR^® ^Green PCR Master Mix (Qiagen), 20 pmol of each primer, and 250 ng of cDNA were added to a final volume of 25 μl. Cycling was performed with the protocol described above. Data acquisition was carried out after each extension step, and a melting curve was performed in 0.1°C steps from 50°–95°C. Real-time RT-PCR efficiencies were calculated from the given slopes in Opticon™ 2 Monitor software. Real-time RT-PCR efficiency rates were high (values of 2.00). Experiments were performed three times in triplicate, the inter-test variation was ≤ 2%, and the intra-test variation ≤ 1%.

## Results

### Light microscopic localization of COMP during human embryonic limb development

**Figure 1:**
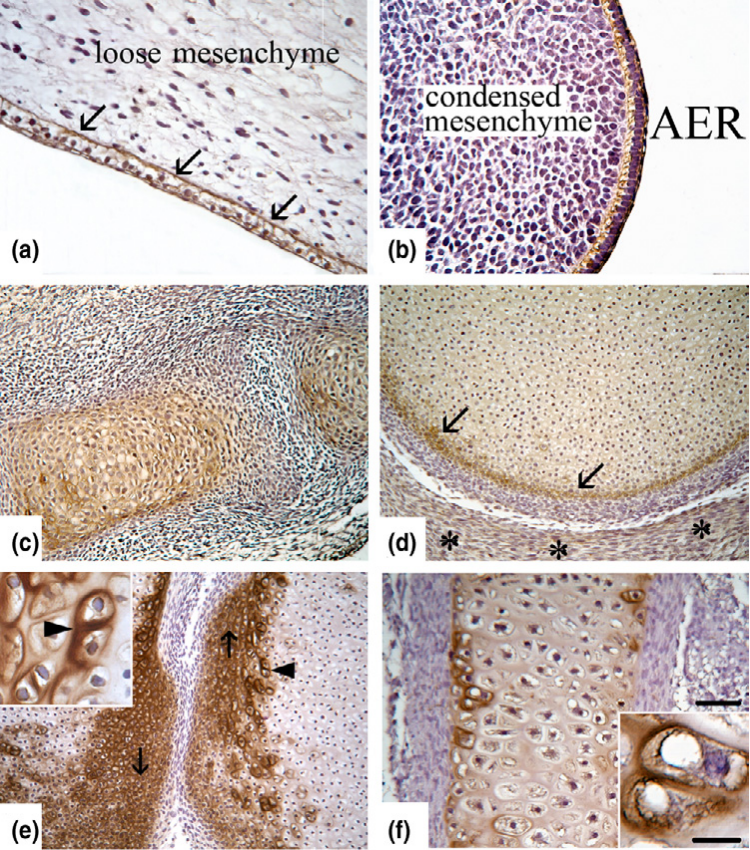
Light microscopic localization of cartilage oligomeric protein (COMP) during early human bone and joint development. **(a) **The basement membrane zone of the dermal-epidermal junction is positive in a human embryo at (gestational week) gw 8 (arrows); the loose mesenchyme is not stained. **(b) **The same is true for the apical ectodermal ridge (AER), the starting point of limb development. Also, the condensed mesenchyme at this developmental stage is not stained. **(c) **At gw 10, the matrix of developing bones is positive for COMP. **(d) **Later, at gw 12, during joint development, COMP staining is restricted to the outer margins of the developing epiphysis (arrows), whereas the developing acetabulum shows still less staining (asterisks). **(e) **Pronounced staining for COMP (arrows) is seen adjacent to the developing joint space. The arrowhead indicates the area from which the high-magnification micrograph was taken (inset). The arrowhead in the inset indicates COMP staining. **(f) **At gw 12, COMP staining is found in the outer regions of the diaphysis and is mainly pericellular (inset). Bars = 70 μm in (f), as for (a)-(e), and 40 μm in inset (f), as for inset (e).

During human embryonic development from gw 8 to gw 12, basement membrane zones of the developing skin stained positive for COMP whereas the mesenchyme remained unstained (Figure 1a). In limb buds, staining for COMP was found in the basement membrane zone of the apical ectodermal ridge (AER), and the condensed mesenchyme was not stained (Figure 1b). During further development of the long bones at gw 10, staining for COMP was seen throughout the extracellular matrix of the cartilage (Figure 1c). Later, at gw 12, staining for COMP became restricted to the margins of the developing epiphysis (Figure 1d), the developing joint surface (Figure 1e), and the diaphysis of long bones. COMP was seen mostly pericellularly around hypertrophic chondrocytes along the edges of the shaft of the diaphysis (Figure 1f).

### Western blot and localization of COMP and its mRNA in healthy and OA human cartilage

**Figure 2:**
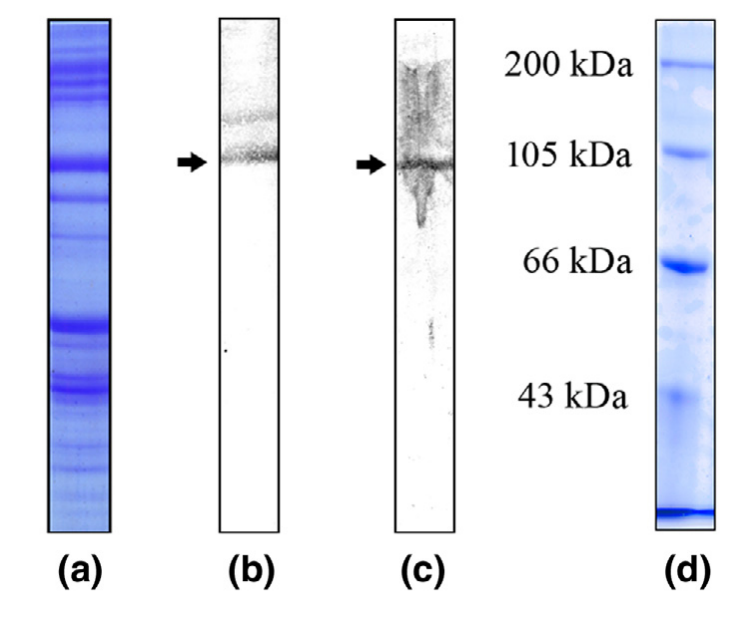
Western blot. **(a) **Coomassie blue staining of the tissue extract of osteoarthritic cartilage taken from the area adjacent to the main defect, **(b) **clear bands at 105 kDa for cartilage oligomeric protein (COMP) (arrow) and a fainter band at 160 kDa in the same extract, **(c) **a clear band at 105 kDa, and a smear seen for healthy articular cartilage and **(d) **shows the molecular weight marker.

**Figure 3:**
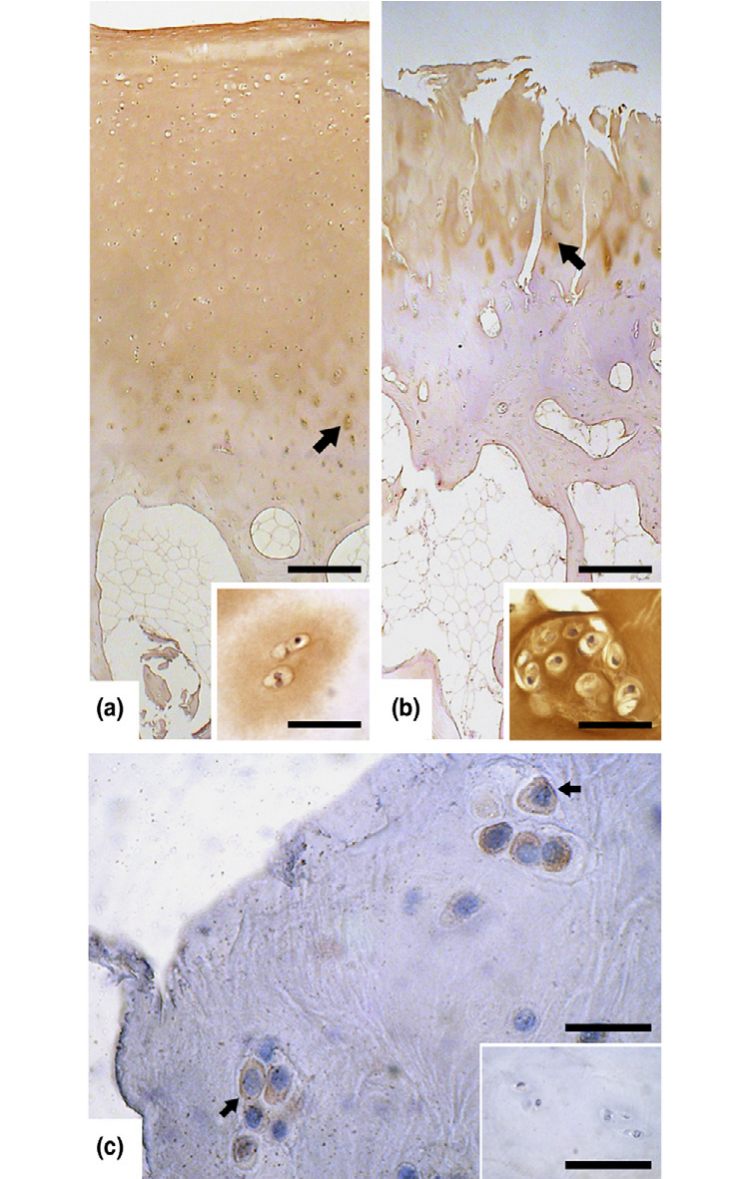
Light microscopic detection of cartilage oligomeric protein (COMP) and its mRNA. **(a) **Staining for COMP is seen in the interterritorial matrix of the superficial and middle zones of healthy cartilage, whereas in the deeper zones a more pericellular pattern is found (arrow and inset). **(b) **In osteoarthritic (OA) cartilage of late disease stages, staining is seen mainly in clusters (arrow and inset). **(c) ***In situ *hybridization of COMP mRNA localizes it mainly in the cytoplasm of chondrocytes found in clusters of OA tissue (arrows); inset depicts a negative control of healthy cartilage. Bars, 70 μm in (a), (b), and inset (c) and 40 μm in (c) and insets (a) and (b).

The anti-COMP antibody [1] cross-reacted with human COMP from healthy (Figure 2, lane 3) and OA cartilage tissue extracts taken from the area adjacent to the main defect (Figure 2, lane 2). The 105 kDa band for a monomer was seen in both extracts, whereas a second band was found only in the OA cartilage sample and might represent a covalently bound binding partner of COMP (for example, one of the matrilins). This phenomenon has been observed with COMP in other instances. The smear in the blot of healthy cartilage tissue (Figure 2, lane 3) probably results from the high aggrecan content, which is missing in OA tissue. This is why this smear is not found in Figure 2, lane 2, where aggrecan is lost (M. Paulsson, personal communication). With the help of light microscopic immunohistochemistry, COMP was localized in healthy knee joint cartilage tissues in the pericellular, territorial, and interterritorial matrix compartments. This was seen in the superficial and middle zone. In contrast, in the deep zone near the tidemark, COMP was found only in the pericellular space (Figure 3a and inset). In OA cartilage, in the area adjacent to the main defect, pronounced staining for COMP was seen (Figure 3b), especially in the pericellular matrix of cell clusters (Figure 3b, inset). With the help of light microscopic *in situ *hybridization, the mRNA for COMP was detected in the cytoplasm of chondrocytes of the superficial and middle zones of healthy cartilage tissue (data not shown) and also in chondrocytes mainly found in clusters in the area adjacent to the main defect in OA cartilage (Figure 3c).

### Immunohistochemistry of COMP in healthy and OA cartilage at the ultrastructural level

**Figure 4:**
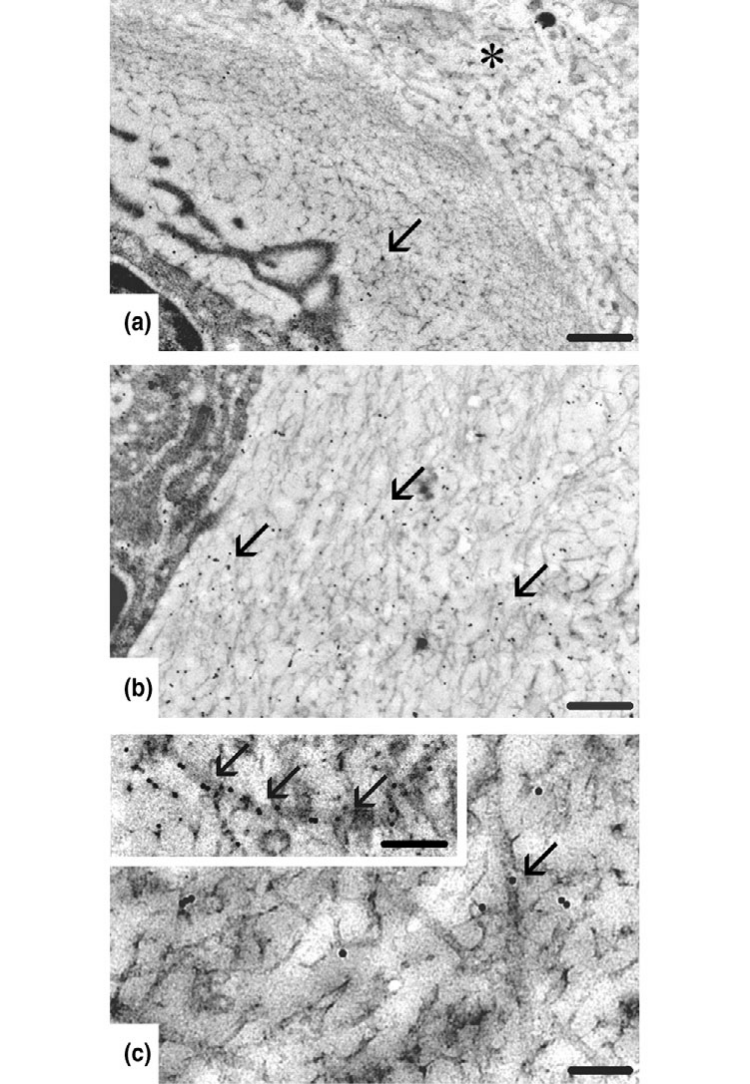
Immunogold histochemistry for cartilage oligomeric protein (COMP) of healthy and osteoarthritic (OA) tissue taken from the area adjacent to the main defect. **(a) **Healthy cartilage tissue with staining for COMP in the pericellular space (arrow) and in the territorial matrix (asterisk). **(b) **The pericellular space of a type 2 cell of OA tissue taken from the area adjacent to the main defect; note the stronger staining compared with the healthy tissue (arrows). **(c) **Higher magnification of the interterritorial matrix from healthy cartilage tissue; note the sparse COMP staining on fibers (arrow). Inset shows higher magnification of the interterritorial matrix taken from the area adjacent to the main defect; note the stronger staining for COMP on fibers (arrows). Bars, 0.4 μm in (a) and (b) and 0.2 μm in (c) and inset.

To elucidate which components in the differing matrix compartments stain for COMP, an ultrastructural analysis was performed. In healthy cartilage specimens, COMP was associated mainly with the fine fibrillar structures in the pericellular space (Figure 4a). In OA cartilage taken from the area adjacent to the main defect from patients in the late stages of OA, an increase in staining intensity was found in the pericellular space (Figure 4b). In healthy cartilage, COMP staining was also found in the territorial and interterritorial matrix (Figure 4c), whereas in OA cartilage specimens, staining for COMP was seen mainly on fibers but also next to them (Figure 4c, inset).

### Ultrastructural *in situ *hybridization of COMP mRNA in OA cartilage

**Figure 5:**
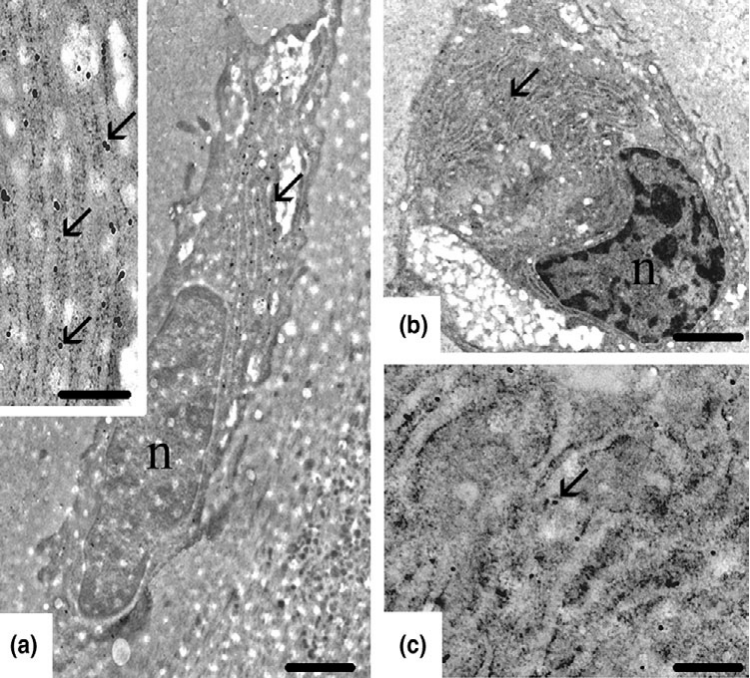
Ultrastructural *in situ *hybridization for cartilage oligomeric protein (COMP) mRNA in samples taken from the area with macroscopically normal appearance of osteoarthritic tissue. **(a) **A type 2 cell is depicted with staining for COMP mRNA (arrows); inset shows a higher magnification. **(b) **Staining for COMP mRNA (arrow) in a type 1 cell. **(c) **Note that the gold particles (arrow) are found only in the cytoplasm adjacent to the rough endoplasmic reticulum. Bars, 0.3 μm in (a) and (b) and 0.25 μm in (c) and inset (a). n, nucleus.

**Figure 6:**
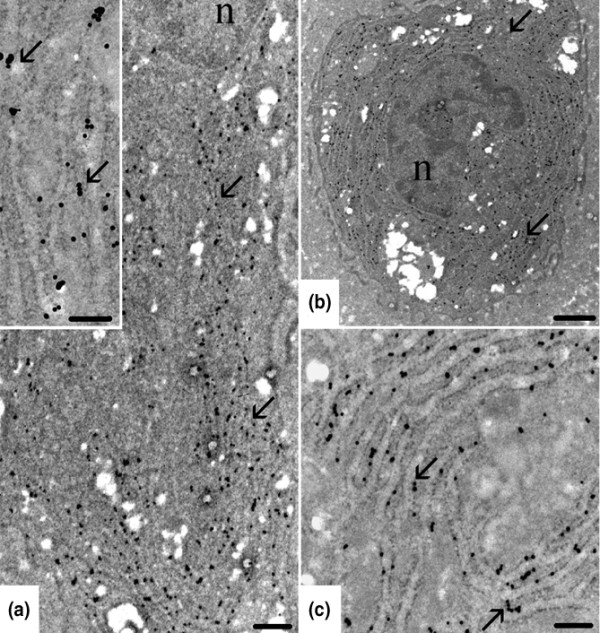
Ultrastructural *in situ *hybridization for cartilage oligomeric protein (COMP) mRNA of the area adjacent to the main defect of osteoarthritic tissue. **(a) **Strong staining for COMP mRNA (arrows) is seen in a type 2 cell; inset shows a higher magnification. **(b) **Strong staining for COMP mRNA (arrows) is seen in a type 1 cell. **(c) **Note that the gold particles (arrows) are found only in the cytoplasm at the rough endoplasmic reticulum. Bars, 0.3 μm in **(a) **and **(b) **and 0.25 μm in **(c) **and inset **(a)**. n, nucleus.

From earlier investigations on the pathogenesis of OA, we are aware of two different cell types found in the late stages of the disease [33,34]. Type 1 cells are the diseased chondrocytes found in regions with a macroscopically normal appearance of the OA cartilage, and type 2 cells are elongated, fibroblast-like cells found mainly in the area adjacent to the main defect. A small number of type 2 cells can also be found in the regions with a macroscopically normal appearance in OA cartilage and vice versa: a few type 1 cells are also present in the area adjacent to the main defect. To elucidate which cells, type 1 or type 2, produce COMP mRNA, we performed *in situ *hybridization at the electron microscopic level. In cartilage tissue with a normal appearance from the lateral aspects of a condyle of the OA patients, COMP mRNA was detected in type 2 cells (Figure 5a and inset) and less staining was seen in type 1 cells (Figure 5b,c). In contrast, in tissue samples from the area adjacent to the main defect of OA cartilage of late stages of the disease, strong staining for COMP mRNA was detected in the cytoplasm of type 2 cells (Figure 6a) and type 1 cells (Figure 6b,c).

**Figure 7:**
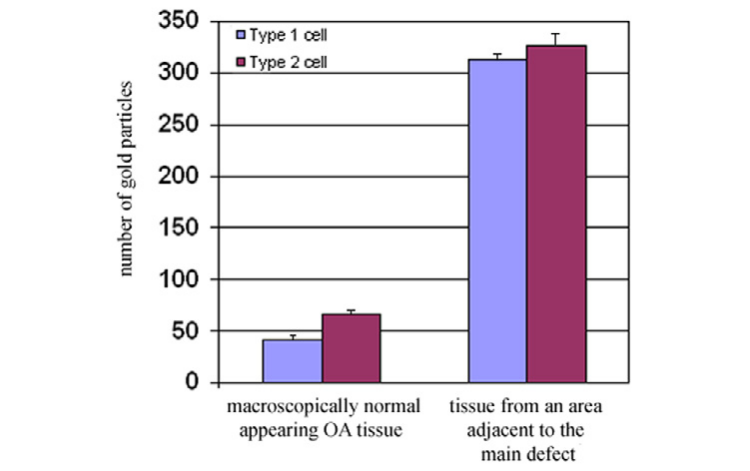
Statistical analysis of the ultrastructural *in situ *hybridization. The two bars on the left depict the mean numbers of gold particles for cartilage oligomeric protein (COMP) mRNA in type 1 and type 2 cells from the area with a macroscopically normal appearance of osteoarthritic (OA) tissue. The two bars on the right show the mean numbers of gold particles in the same cell types taken from the area adjacent to the main defect of OA cartilage.

The number of gold particles detected in the samples with a macroscopically normal appearance from OA tissue revealed staining intensities of approximately 42 (SEM = 3.4) in type 1 cells and 66 (SEM = 4.1) in type 2 cells. This represents a significant difference (*p *≤ 0.01). In contrast, in both cell types found in the areas adjacent to the main defect of OA tissue, approximately 320 gold particles (SEM = 13.4) were detected (Figure 7). This represents a statistically significant (*p *≤ 0.01), approximately 83% difference in staining intensity for the cells taken from the two areas.

### Quantitative real-time RT-PCR

**Figure 8:**
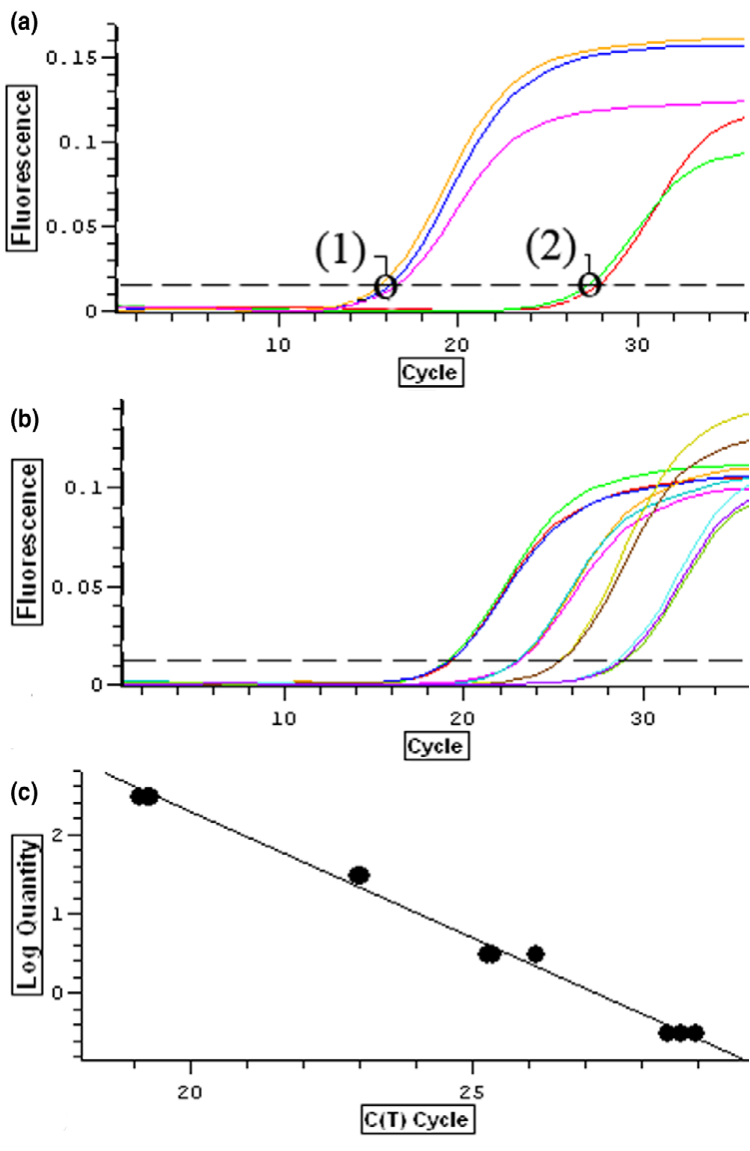
Quantitative real-time reverse transcription-polymerase chain reaction (PCR). **(a) **Graphs for cartilage oligomeric protein (COMP) of samples of osteoarthritic cartilage tissue taken from the area adjacent to the main defect (1) and of cartilage tissue with a macroscopically normal appearance (2). Note that the slopes of the graphs, each color representing one PCR reaction, are highly similar. A significant difference between threshold cycle [C(T)] values of (1) and (2) is shown. **(b) **The decreasing C(T) values of the standard dilution of the housekeeping gene *HPRT-1 *are shown. **(c) **Standard curve derived from the standard dilution.

To validate the semi-quantitative results from the ultrastructural *in situ *hybridization, we performed quantitative real-time RT-PCR. The mean threshold cycle value for COMP cDNA detected in tissue samples from patients with late stages of OA taken from the area adjacent to the main defect is 16.2, representing a relative ratio of 8.28, and the value detected in samples of cartilage tissue with a macroscopically normal appearance is approximately 27.5 (Figure 8a), representing a ratio of 0.16. The relative ratios were calculated according to the algorithm of Pfaffl. The relative ratio for COMP in normal cartilage tissue is approximately 98% lower when compared with OA tissue. The calibrator curve obtained by the correlation of the threshold cycle values with the dilution series of the housekeeping gene exhibited a low (≤ 1%) intra-test variation (Figure 8b,c). The validity of the PCR results was confirmed by sequencing and by the melting curves performed for each PCR (data not shown).

## Discussion

Until now, nothing has been known about the role of COMP during human development. COMP has been shown to be located in porcine joints, where high levels were seen in the proliferating zones and low levels were seen in the hypertrophic zones [5], which differs from what we found for human embryonic development. During human bone development investigated here, the strongest staining for COMP was seen in areas where joint development had taken place. This differs from mouse development, in which COMP is seen mainly in the perichondrium, but is in line with the present results, which demonstrate COMP-positive hypertrophic cartilage zones also during human development [27]. We were able to show COMP-positive superficial cartilage zones, as already described for mice [24]. Additionally, we detected COMP in the middle zones and in deep cartilage zones near the tidemark. Furthermore, COMP was detected in the basement membrane zones of the AER, the earliest signs of limb bud formation, but not in the condensing mesenchyme as described for murine development [24]. There is evidence from *in vitro *models that COMP is involved in the regulation of chondrogenesis [25]. In contrast, COMP knockout mice do not exhibit an obvious skeletal phenotype [26]. In light of these previous results and the localization of COMP during human limb development in the correct spacial and time relationship presented here, which is different from the more general distribution of COMP during mouse development, it can be speculated that COMP plays a more specific role during human skeletal development, especially in joint formation, which needs to be further elucidated.

COMP is also present in healthy adult articular cartilage, as demonstrated here with the help of a Western blot, as well as *in vivo *at the light and electron microscopic level. Earlier, COMP was detected in the normal growth plate of primates [35] and was shown to bind to adult normal bovine chondrocytes *in vitro *[8]. COMP was also shown to bind to matrilins [9], as well as to collagen types I, II, and IX [11]. This could imply that the protein could function as one of the link molecules to organize and stabilize the extracellular cartilage matrix. Indeed, at the ultrastructural level, COMP was found to be associated with the fibers of the pericellular, territorial, and interterritorial space of healthy and OA human cartilage tissue taken from the area adjacent to the main defect. Furthermore, COMP staining was also detected next to the cells in the pericellular space associated with its fine fibrillar material. Therefore, COMP might also be involved in chondrocyte regulation, as is already known, for example, for decorin [34].

It has been shown that high serum levels of COMP are associated with the progression of OA [21]. Altered cell-matrix interactions underlie the pathogenesis of OA [36], especially for late disease stages investigated here [34]. The process of OA seems to begin with a continuous breakdown of the matrix framework [37] and results in a loss of matrix strength [38]. Here we found increased amounts of COMP mRNA in the area adjacent to the main defect of OA cartilage of late disease stages, where the main regeneration efforts take place [39,40]. The type 2 cells from this area are the only cells newly emerging in late stages of the disease and are signs of the regeneration processes [34,39,41]. They produce five times more COMP mRNA than the same cells taken from the tissue with a macroscopically normal appearance of the lateral aspects of a condyle of OA cartilage. Furthermore, these results were backed up by the quantitation of real-time RT-PCR results. Dynamic loading increases the expression of COMP, and higher COMP mRNA levels can be found two days after compression [14]. This is in line with the present results demonstrating the highest COMP mRNA levels in the regions adjacent to the main defect, where the highest load occurs. This can be taken as evidence that COMP, with its multiple binding possibilities, might be secreted by the chondrocytes in late stages of the disease to ameliorate the breakdown of the extracellular matrix. An enhanced production of matrix components at the transcriptional and translational levels has also been demonstrated for other molecules with known functions within the matrix framework, such as decorin and biglycan [33] or perlecan [41], whereas the main cartilage collagen, collagen type II, has been shown to be downregulated [42].

One of the known factors of *COMP *gene expression regulation in mice is the LRF, which inhibits *COMP *transcription and decreases collagen type II expression via downregulation of bone morphogenetic protein-2 *in vitro *[15]. The human *COMP *gene promoter contains a typical consensus site for binding to LRF/factor binding inducer of short transcripts protein-1 (FBI-1) [15]. If FBI-1 [43] acts as human counterpart of murine LRF, human *COMP *expression should be downregulated by FBI-1. As shown here, in late stages of human OA *in vivo*, chondrocytes upregulate their *COMP *expression and, as shown earlier, downregulate their collagen type II expression [42]. This differs from the mouse model that indicates that LRF/FBI-1 is the general transcription factor for the downregulation of *COMP *and collagen type II [15]. If LRF/FBI-1 initially downregulates *COMP *and collagen type II in human OA, which in turn enhances the matrix breakdown and thereby increasing the mechanical load of the diseased tissue, this mechanical load could counteract the LRF/FBI-1 effect and upregulate only the *COMP *expression in late stages of the disease, as shown here for the areas bearing the highest load in human OA tissue *in vivo*.

## Conclusion

In summary, our results demonstrate that COMP is present in the earliest stages of human bone and joint development. COMP is also a component of the adult healthy articular cartilage matrix and is produced by the chondrocytes. Furthermore, we were able to show that during late stages of OA, increased amounts of COMP are produced by type 1 and type 2 cells in the area adjacent to the main defect and that due to its wide binding repertoire, COMP might therefore be involved in the regeneration efforts of OA cartilage tissue as a factor secreted by chondrocytes to ameliorate the matrix breakdown.

## Abbreviations

AER = apical ectodermal ridge; COMP = cartilage oligomeric protein; DIG = digoxigenin; FBI-1 = factor binding inducer of short transcripts protein-1; gw = gestational week; IgG = immunoglobulin G; LRF = leukemia/lymphoma-related factor; OA = osteoarthritis; PBS = phosphate-buffered saline; RT-PCR = reverse transcription-polymerase chain reaction.

## Competing interests

The authors declare that they have no competing interests.

## Authors' contributions

TSC performed the immunohistochemistry and *in situ *hybridization of the normal and OA cartilage. MK is responsible for the Western blots. SK and NM are responsible for the real-time PCR and the overall editing of the manuscript. All authors read and approved the final manuscript.
